# Online social media poses opportunities and risks in autistic youth: implications for services from a qualitative study

**DOI:** 10.3389/fpsyt.2023.959846

**Published:** 2023-06-30

**Authors:** Phil Wai Shun Leung, Shirley Xin Li, Eleanor Anne Holroyd, Carmen Sze Oi Tsang, William Chi Wai Wong

**Affiliations:** ^1^Department of Family Medicine and Primary Care, LKS Faculty of Medicine, School of Clinical Medicine, The University of Hong Kong, Hong Kong, Hong Kong SAR, China; ^2^Haven of Hope Christian Service, Hong Kong, Hong Kong SAR, China; ^3^Department of Psychology, University of Hong Kong, Hong Kong, Hong Kong SAR, China; ^4^Faculty of Health and Environmental Sciences, Auckland University of Technology, Auckland, New Zealand

**Keywords:** online social media, autism, qualitative research, young adults (18–29 years), social functioning, online scam, internet safety

## Abstract

**Background:**

Autistic people are vulnerable to developing mental health problems due to their difficulties in managing social situations and interpersonal relationships. The popular online social media (OSM) can be a potential solution to these concerns for their social lives as it allows non-face-to-face social interactions, however it remained unclear how this group is using these online platforms. This study explored their experiences of using online social media, and their perceived benefits and risks associated with this use.

**Method:**

Qualitative data was collected through in-depth face to face interviews. We recruited 13 autistic young adults who were with normal intelligence from two community centers in Hong Kong. Interviews were conducted in a semi-structured format by experienced clinical psychologist and social workers.

**Results:**

Four themes were identified from the data, namely: (1) Paradox of using OSM to supplement social needs; (2) Unpleasant social interactions in the online environment; (3) Restricted and repetitive pattern of interest leading to troubles in OSM use, and; (4) Privacy and personal safety issues. The results suggested that while OSM could be a useful tool for our participants to connect with existing friends and to meet new ones, their limitations, such as difficulties in understanding languages and social interaction as well as restricted patterns of interests could potentially put them at risk, including interpersonal conflicts, cyber-bullying, financial scams, as well as unwanted sexual experiences. These experiences not only paradoxically increased their sense of loneliness and their distance from others, but also resulted in a negative impact on their mood and functioning.

**Conclusion:**

This qualitative study provided some insights into the OSM use among autistic young adults. OSM could be a useful tool to overcome potential limitations in social interactions, but at the same time it could potentially bring detrimental consequences. In order to maximize the benefits of online social networking, there is a need for timely guidance and support to use OSM among autists, and for psychoeducation to promote awareness, and enable adequate skills and behaviors attainment associated with safe OSM use.

## Introduction

Autistic people show significant impairments in social communication and interactions. Among those with normal intelligence, their repeated failures in social situations make them vulnerable to developing various psychological problems, such as depression, anxiety disorders, mood swings, self-harm behaviors, and suicidal ideations and behaviors (1–3). Whitehouse and colleagues reported that the perceived loneliness and poor quality of friendships among autistic people were significantly correlated with mood symptoms (4). A population-based cohort study conducted among autistic late adolescents suggested that impaired social communication was associated with a higher risk of suicidal ideations and plans (5).

Recent research has explored the use of online social media (OSM) among autistic people without intellectual disability, and how OSM could improve their quality of social life and psychological health. OSM refers to any online platforms such as WhatsApp, Facebook, Instagram, Twitter and some dating apps that allow or even facilitate social interactions between people irrespective of their locations. When compared to face-to-face encounters, OSM requires fewer social demands and interpretations on non-verbal communication, allowing more time to read and process words, and providing higher control on the pace of communication (6, 7). Therefore, OSM presents a comfortable setting for autistic people to interact with other people while lessening inherent anxiety arising from their autism.

OSM has been found to be a useful platform as a means of education, communication, as well as emotional displays among a group of health care professionals (8). It has also become a popular social networking tool among autistic people in recent years (9–12). According to two studies conducted in the United States, the proportion of autistic people who used OSM reached 80% (9, 12). Another study carried out in Netherland showed that autistic adults spent significantly longer time on OSM (13.95 h per week) when comparing with neurotypical peers (9.01 h per week) (10). Although van Schalkwyk and his colleagues found that the proportion of using OSM among autistic adolescents was lower than that among non-autistic, the figure was still high which reached nearly 70% (11). Given the heavy use of OSM among this group, the experience of using OSM can bring a profound impact on their social and overall quality of life.

Positive impacts of OSM on the social lives among autistic people have been reported in some previous research. Kuo and colleagues found that those who used social networking programs reported a greater sense of security in their friendships than those who did not; and, those who visited websites for finding or maintaining relationships also reported more positively orientated friendships (13). Significant positive relationships between OSM and friendship quality among autistic adolescents has also been reported (9, 11). These results support the ‘increase hypothesis’ (14), which means that Internet can provide extra opportunities for interaction in addition to offline communication, which, in turn, facilitates relationship building. Another study also found that increased Facebook use among autistic adults was associated with a higher level of happiness, although this association did not persist at extreme levels, implying that happiness would plateau when Facebook was excessively used (12).

Despite these positive impacts, the risks associated with OSM use cannot be ignored. Even for neurotypical people, OSM users may face frauds or scams for money, unwanted sentimental engagement or even unwanted sex. Mesch and Dodel suggested that the occurrence of an online fraud required two processes (15), namely the attempts made by offenders to approach victims and the active risk behaviors engaged in by the victims, such as responding to requests and providing certain personal information. For autistic people, their difficulties in ‘deceiving others’ (i.e., hiding some of their true personal details) and understanding hidden social rules or culture may result in an inability to protect themselves in the online environment, and accordingly put them at heightened risk of being the victims of frauds (6). A recent survey conducted with autistic adults in England found that 31% of them had experienced cyberbullying victimization, 2% had engaged in cyber-aggressive behaviors, and a further 8% reported both cyberbullying victimization and acted as cyber-aggressors (16). Another survey in Taiwan found the prevalence of cyberbullying victims and perpetrators in autistic adolescents who were with normal intelligence were 14.6 and 10.0%, respectively (17). Experiences of cyberbullying victimization was reported to be associated with increased symptoms of anxiety among autistic youths (18).

Although there is no local statistics regarding the prevalence of mobile phone use among autistic people to date, official statistics suggested that more than 99% of the adolescents and adults in Hong Kong had a smartphone (19). The popularity of OSM has also been rapidly increasing in recent years, especially among adolescents and young adults who spends nearly 20 h per week on online social activities according to an official statistic released in 2021 (20). These figures were in line with the aforementioned Western findings, suggesting heavy OSM use among young adults no matter they are neurotypical or autistic.

The current research was the first Hong Kong based study that aimed to provide a detailed investigation of the experiences of OSM use, and the associated benefits and risks among autistic young adults who were with normal intelligence. Interview was thought to be an effective means of data collection to capture detailed voices and ‘state-of-mind’ among a specific group of population (8, 21). As a result, this study employed a qualitative study design as the data collection strategy, which allowed a detailed investigation of this new phenomenon. The key objective was to promote safe OSM use among autistic young adults, the findings were anticipated for use in designing tailor-made interventions to enhance risk awareness and promote self-protection for this special group.

## Methods

The present study aimed to explore the experience of OSM use among autistic young adults in Hong Kong through in-depth face-to-face interviews. Data collection was carried out using semi-structured individual interviews. The questions were designed with the consensus of all investigators covering the major issues from the literature concerning OSM use among our participants, which are described in Appendix 1.

### Participants and sampling

**Figure 1:**
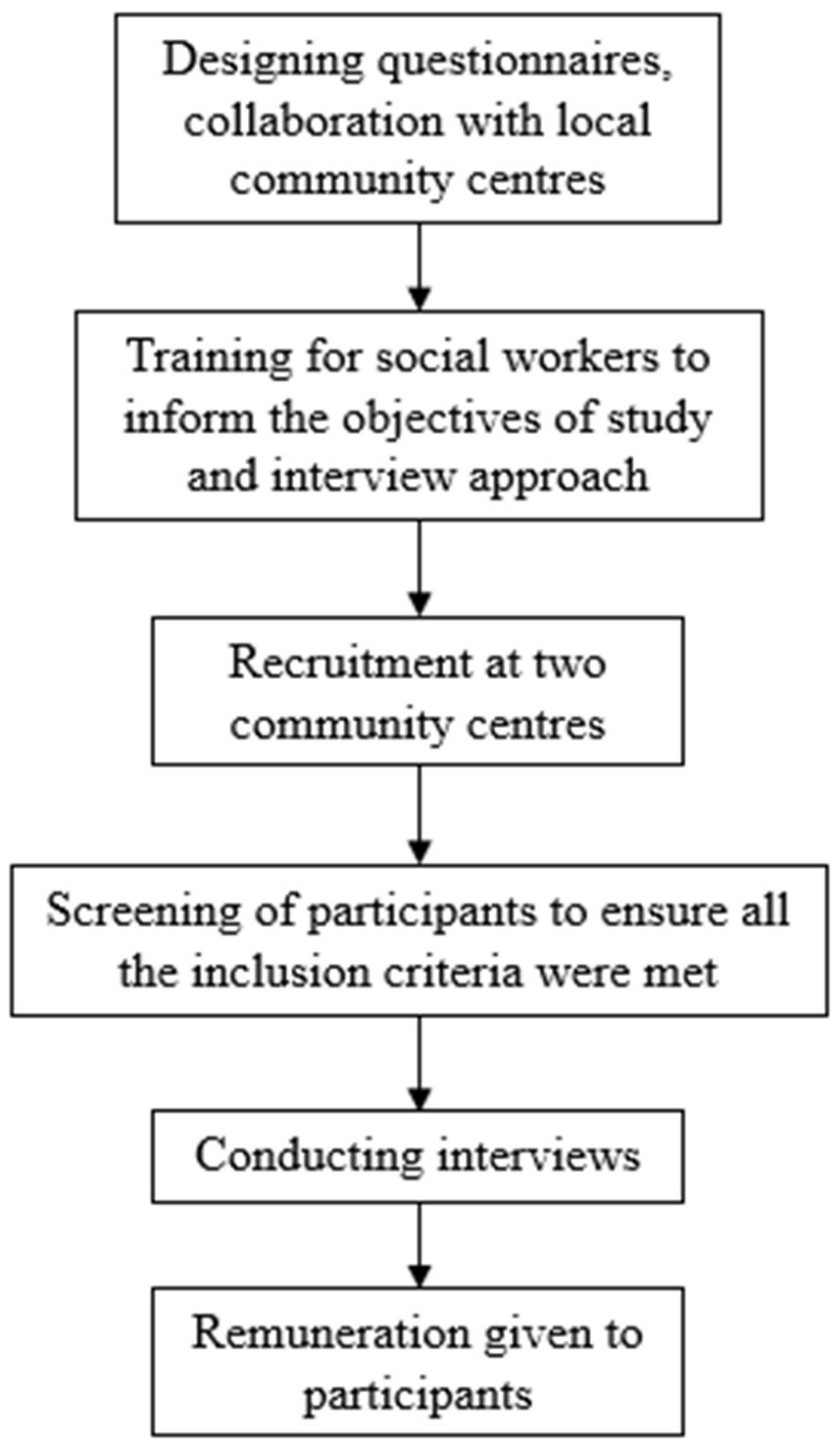
Procedures of subject recruitment.

Procedures of participant recruitment was summarized in Figure 1. Recruitment was conducted in collaboration with two local community centers: Haven of Hope Tseung Kwan O & Sai Kung District Support Centre (DSC), operated by the Haven of Hope Christian Service, a non-governmental organization in Hong Kong. The DSC, serves more than 1,100 members, providing multi-disciplinary services for people with physical or intellectual disabilities and their caregivers in order to assist them to live independently in the community. Autistic people are one of the target service user groups in DSC. Another NGO, ‘Color My Sky – Support Centre for Persons with Autism’ (CMS) under Yang Memorial Methodist Social Service, also participated. This is a community-based center under Hong Kong Social Welfare Department, serving autistic adolescents and young adults with normal intelligence to support their transition needs when entering adulthood.

Inclusion criteria included young adults, aged between 18–29 years old, who were with normal intelligence (IQ score of 70 or above) and had received a diagnosis of autism spectrum disorder. The interviews were conducted in Cantonese as that was most the commonly spoken language in Hong Kong, and the language of choice for the interviews. Another inclusion criterion was previous experience of using OSM. A screening question ‘Have you ever used online social media in the past?’ was asked before they were invited to participate in the interview. Those with comorbid diagnosis of psychotic disorders were excluded. Recruitment would be carried out until saturation of information was reached when no further new codes or themes was expected to be found in subsequent interviews.

### Procedures

The interviews were conducted by either a clinical psychologist or social workers. The male clinical psychologist was the principal investigator of this research with more than 10 years of experiences working with this population, while the social workers were case workers of the participants (relevant ethics described below) and had worked with the participants for at least 2 years. Rapport and enhancing comfort were crucial elements when conducting interviews with autistic people (22). For some participants who had a high level of social anxiety in front of strangers, their case workers would accompany them in the interviews in order to make them feel comfortable to express themselves. Nonetheless, the nature of voluntary participation was stressed when the participants were invited to join (see Ethics part for details). All social workers were trained and informed of the details of this study and interview approaches before conducting an interview. As a token of appreciation, remuneration in the form of a supermarket coupon valued at HK$50 was provided to each participant who had completed the individual interview.

All interviews were conducted in one-to-one format. Ten of them were carried out in a quiet room of the center from where they were recruited. Given that this study was conducted during COVID-19 pandemic and face-to-face interviews were not always feasible, the remaining three interviews were conducted through Zoom. The duration of interviews was varied depending on responsiveness of participants and most lasted for 30 min to one hour. All the interviews were recorded for future transcription.

### Ethical consideration

Given their adequate cognitive ability, informed consent was directly sought from the participants while the parents of the participants were also informed of the objectives, ethics and procedures of the study. Ethical principles were explained including the nature of voluntary participation and their right to withdraw from the study at any time if they wished. They were also assured that non-participation would not affect the quality of service they received from the centers. All information was kept strictly confidential and stored in computer with an encrypted password only be accessible by research staff. In addition, no identifiable personal data would be reported and only aggregate data would appear in future research reports or publications. While there was no foreseeable harm to the participants, parents could seek help from the social workers or clinical psychologist for further individual support or professional assistance in case their children showed any mood fluctuation after their participation of the research.

Ethics approval was obtained from the Institutional Review Board (IRB) of the University of Hong Kong/Hospital Authority Hong Kong West Cluster (Ref no: UW 19-871).

### Data analysis

All the interviews were voice-recorded. The recordings were verbatim transcribed into words manually by research support team, and then imported to the Nvivo software to facilitate the organization of data and subsequent generation of codes and themes. The procedures of analysis followed the steps of thematic analysis suggested by Braun and Clarke to ensure its trustworthiness (23, 24). First, data was reviewed several times by the research team in order to get familiar with the data and identify relevant ideas. Second, these relevant ideas were reviewed and grouped according to their natures and frequency, and initial codes were developed from the transcribed text. Third, codes were reviewed again and related codes were further organized manually which formed different themes. The themes identified formed new concepts about the current use of online social media and effectiveness, risks perception of online frauds and the useful safety precautions among autistic young adults. Fourth, the relationships between each theme were explored which generated a ‘thematic map’ among the entire data set. Finally, the themes were named based on their unique natures, which became the final findings of this analysis. These analyses were intended to inform practical suggestions to design an effective intervention program in order to promote the safe use of OSM among local autistic young adults.

### Rigor

Specific measures have been implemented to ensure the rigor of methodology, namely credibility, dependability, confirmability and transferability (25). To ensure creditability, all interviews were conducted by the principal investigator (clinical psychologist), or by experienced social workers who were familiar with the participants and had received staff training regarding the details of the present study. To achieve high reliability of findings, an audit trail was conducted, and the use of qualitative data analysis software, NVivo, enabled the tracking and storing of all the raw data, coding, as well as the procedures and decision-making during analysis. In addition, more than one data collection site (DSC and CMS) were used to maintain confirmability. Finally, thick and rich description of findings, including direct quotes from participants, are presented in the results below in order to ensure the transferability of data.

## Results

**Figure 2:**
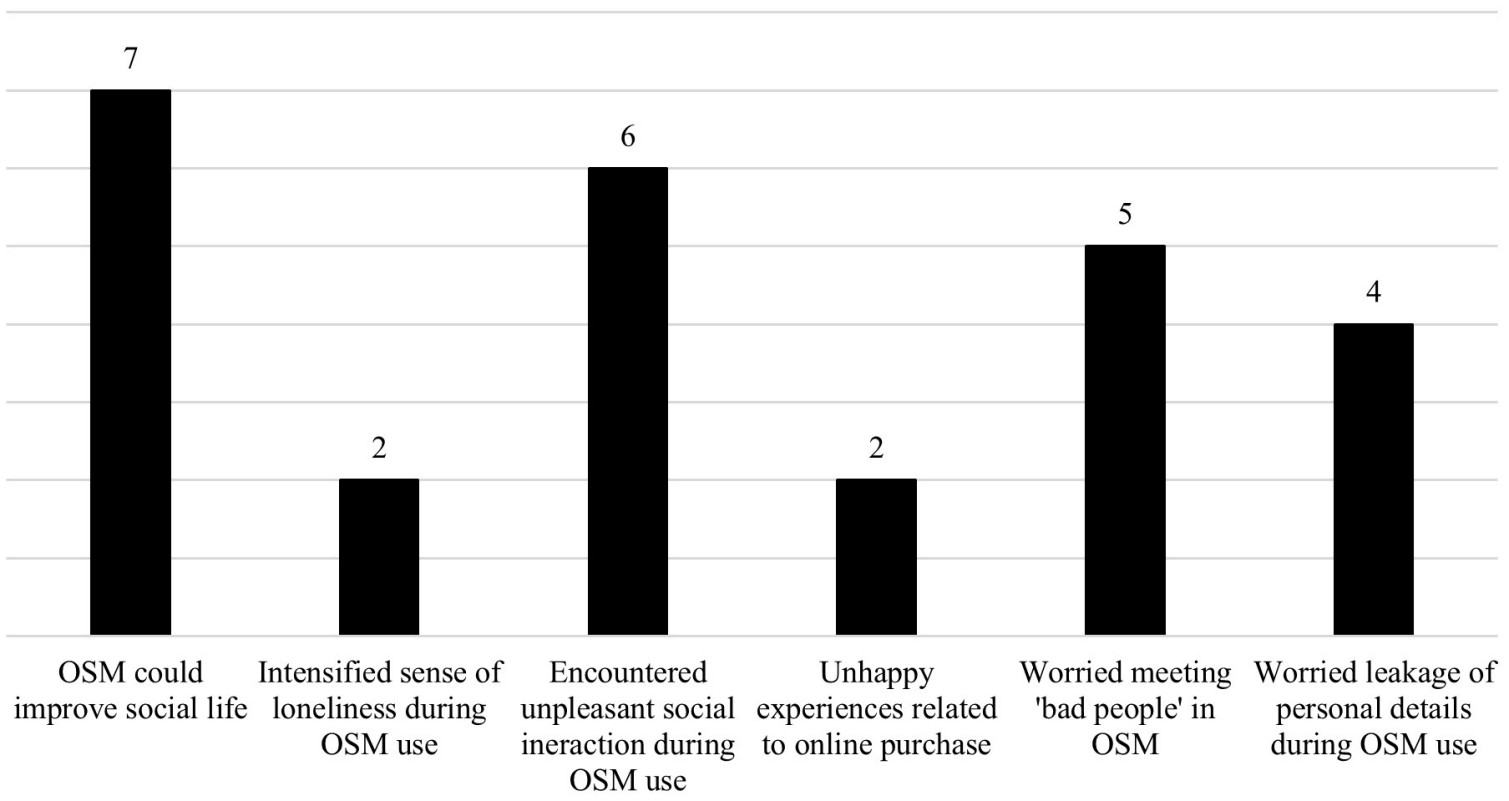
Thoughts underlying OSM use among participants. Numbers denotes the number of participants who expressed the specific thought.

A total of 13 participants were recruited from the two sites, 6 from DSC and 7 from SPA. There were 7 males and 6 females, with an average age of 20.77 years (SD: 2.80 years) and a range of 18–28 years. The demographic details of and the OSM platforms used by these participants are shown in Table 1. The findings of these individual interviews were organized into five main themes, namely: (1) Paradox of using OSM to supplement their social needs; (2) Unpleasant social interactions in the online environment; (3) Restricted and repetitive pattern of interest leading to troubles in OSM use, and (4) Privacy and personal safety related to OSM. Some salient thoughts underlying these themes and their attitudes toward OSM were presented in Figure 2.

**Table 1:** Demographic details of and the OSM platforms used by participants (*N* = 13).

		*n* (%)
Age	18–21 years old	10 (76.9%)
	22–25 years old	2 (15.4%)
	26–29 years old	1 (7.69%)
Gender	Male	7 (53.8%)
	Female	6 (46.2%)
OSM platform used	Whatsapp	10 (76.9%)
	Facebook	9 (69.2%)
	Instagram	9 (69.2%)
	Signal	3 (23.1%)
	Snapchat	2 (15.4%)
	Youtube	1 (7.69%)
	Telegram	2 (15.4%)
	WeChat	2 (15.4%)
	MeWe	1 (7.69%)
	Other dating apps (e.g.*, MeWe, Omi, Tantan, Coffee meets bagel, Base*)	1 (7.69%)

### Paradox of using OSM to supplement social needs

Eight of 13 participants mentioned that they would communicate with their current friends on OSM. Especially during the COVID-19 pandemic, they got updates and saw photos of their friends through various OSM platforms, as well as connecting with their friends using WhatsApp groups when they were not able to meet up face-to-face.

Besides connecting with their own friends, five participants reported that they had made new friends through OSM in the past, and three of them were open to developing an intimate relationship with friends whom they met online. About half of the participants (7 out of 13) thought that OSM was useful for them to improve their social life. They thought that OSM could let them meet other people with similar interests online. For example, they could join some special interest groups that were developed for people with a common interest, such as bus, railway or online game fans groups, which were also popular within ASD population. In addition, they found it easier to maintain a new friendship with the help of OSM. One participant mentioned that once they exchanged their Instagram or Facebook account during their first encounter with friends, it would be easy for them to keep contact and eventually build up friendships.

‘Most of the time after you meet a new friend, it is difficult to see each other again, but once we exchange our OSM account details, this relationship can continue… that means even though we don’t see each other in person again, it appears that we have marked him/her as a friend, so OSM was of a great help’ (007)

A male participant reported that he was found it more comfortable to express his inner thoughts in an online environment and disclose his unhappy incidents, which he was unable to share with others in real life social situations. This could particularly be helpful for autistic people who experienced significant anxiety and difficulties in real life social situations.

‘After we got the contact and communicated through WhatsApp (with friends), we talked with each other frequently… sometimes I could even share my unhappy feelings with them through WhatsApp’ (002)

Among autistic young adults, loneliness resulting from failures in real-life social relationships was a major driving force to seek new friendship through online engagements. One female participant mentioned her lack of interpersonal skills and anxiety in real life social situations made it difficult for her to make new friends. The feeling of loneliness was further intensified due to her poor relationship with family. These frustrating experiences motivated her to seek new friendship through virtual means.

‘When I was young, I could spend 8-9 hours a day on OSM, probably because I did not have many friends, and I felt shy when I was in (real life) social situations… also, it was because I did not like my family as well…’ (006)

Another female participant thought that it was possible to find ‘true love’ using OSM. In the interview, she shared her interactions with her boyfriend, whom she first met through dating apps and subsequently developed an intimate relationship.

‘Some people (they met through online) are kind, for example, when I woke up late for school, he (her boyfriend whom she met through dating apps) would call me and wake me up’ (013)

While some participants viewed OSM as an effective tool to broaden their social network, others held an opposite view. Six of them thought that OSM could not help them to make friends and expand their social networks. Of note is that one female participant commented that OSM was helpful to build up friendship with those people who only wanted to have a casual relationship, rather than a long-lasting one.

Yet, using OSM may not necessarily mean that it could effectively increase the number of friends and reduce one’s sense of loneliness for autistic young adults. Some participants reported that it could sometimes reinforce their sense of isolation from their friends or peers. Through their use of OSM, they were more aware of their distance from others, which further intensified their sense of loneliness which they often experienced in their real lives. For example, the same female participant mentioned when she browsed through her friends’ updates or pictures shared in their OSM accounts, she realized that she was not invited to join the social gatherings of her peers. In this way social media could ‘visually’ show the rejection from her friends and the gap between them.

‘I always saw my friends having so many gatherings (in Facebook), but most of the time I was not invited… I think that I cannot mix well with them, and I feel lonely and weird… sometimes I think that OSM visualizes the gap between my friends and me, especially the “social gap”’ (006)

Another participant mentioned he had made some new friends and started chatting with each other in OSM in the past. However, he eventually found out that they had some underlying intentions such as getting money from him or persuading him to sign an insurance deal. These experiences not only triggered his unpleasant feelings, but also made him feel unable to trust any friends he met online.

‘(Making friends through OSM) is unpleasant… I had met a few strangers who asked if I could be their friend in OSM (Facebook)… they added me, and we chatted for some time… but finally I realized that they were actually trying to persuade me to sign deals of insurance or skin-care treatments, they had a purpose… these experiences were very unpleasant” (007)

The data reflect that while some autistic young adults hoped to broaden their social network through the use of OSM, it could paradoxically reinforce their sense of loneliness and reflected their failures in their real social lives.

### Unpleasant social interactions in the online environment

Among our participants, nearly half (6 out of 13) reported to have experienced unpleasant social interactions while using OSM and felt that their mood was affected when they engaged in online spats or arguments with friends or other users. For example, a female participant experienced cyberbullying when she was ‘unfriended’ by her peers after she had posted some negative comments toward their favorite Korean pop singing group. She experienced significant stress and mood fluctuations after exchanging some provocative messages with her online friend.

‘I have said something wrong before (during Facebook use), such as saying bad words towards other (K pop) idols… it irritated my friends and they did not reply to me any more. So after this incident, I did not post any comments at all, and not talk to other friends any more… and I did not want to make any friends through online platforms again, I felt very stressful…’ (001)

The impact of these unpleasant events could be traumatic given that autistic people, regardless of their intellectual abilities, tended to ruminate and find difficulty in overcome what they have gone through. The same female participant mentioned explicitly that she had spent a long time to recover from her past frustrations arising from conflicts with other OSM users. She actively avoiding share what these unhappy experiences were on interview as she did not want to recall these memories and re-experience further mood disturbance.

Interviewee: ‘Yes… I don’t want to meet the friends who I knew through OSM any more, it is too stressful to me… I tried it before (in Facebook) but I won’t do it again … as my mother has banned me from meeting any online friends, she thought it was very dangerous…’

Interviewer: ‘Would you mind sharing your experience?’

Interviewee: ‘Err … I don’t want to talk about it again, I spent such a long time to forget it…’ (001)

Even when some participants had not experienced any conflict online personally, three of the participants mentioned that they felt frustrated when they had ‘witnessed’ other OSM users quarreling with each other on the OSM platforms. For example:

‘When I saw the posts… other users were arguing with each other, I felt a little unhappy and upset’ (003)

The difficulties in emotion recognition, a common limitation of autistic people, could influence the effectiveness of communication through text messages during their OSM use. Although they did not need to interpret the facial expression of their friends which was necessary in real life, the presumably less complicated *emoji* at times posed confusion for them. One female participant mentioned that she was only able to understand the surface meaning of words but not the underlying tone or mood or *emoji* expressions. These misunderstandings, in turn, became an obstacle for her to build up on line friendship in depth and created further conflict with her friends.

‘I cannot communication well with my friends… maybe there are so many emoji expressions in our WhatsApp group, it is very confusing, so it creates misunderstanding’ (009)

Engagement with OSM resulted in a detrimental incident to one participant. This participant had experienced unwanted sexual behaviors “with a friend she knew” through OSM in the past. She mentioned that she had wanted to build up a new relationship through online because of the repeated failures in her ‘real’ social life. She described this incident as a serious lesson for her, leading to her feeling she could not establish high levels of trust toward any online friends in the future. She has stopped searching for and developing any intimate relationships through OSM platforms or dating apps.

‘I had met some bad people through online dating (in dating apps) in the past, and I dare not to have close contact with them any more… I could not have a high level of trust towards anyone I met in OSM, it was because what had happened to me when I was young, at my 19 years of age’ (006)

These findings suggested that the use of OSM did not appear as an ideal solution to address difficulties in social situations among local autistic young adults. Using OSM may simply transfer their difficulties from face-to-face to online context.

### Restricted and repetitive pattern of interest leading to troubles in OSM use

Highly restricted and fixated interests and behaviors were one of the core symptoms of ASD according to the Diagnostic and Statistical Manual of Mental Disorder, fifth edition (26). Our findings suggested that these symptoms could make autistic people more vulnerable to unpleasant experiences during their OSM use. Their fixated interests, which were of abnormally high intensity, was a crucial underlying factor that triggered conflicts with other online users. According to the female participant who had strong preoccupation with K-pop, conflicts occurred when she read critical comments targeting this group, as well as when she wrote similar attacking posts toward other K-pop groups. The latter caused her friends to ‘unfriend’ her, which made her feel very upset and remorseful.

Another concern arising due to the fixated interests of autistic people was their online purchasing behaviors through OSM. This preoccupation may potentially give rise to their excessive online shopping without considering the potential risks. For example, they may come across sellers who promoted their products in various OSM platforms or discussion forums created by other people with similar interests. Two participants reported getting frustrated by online shopping through this means. A female participant mentioned her past experiences of financial scam, such as not getting the products after paying money, as well as paying extra money for the delivery cost.

Interviewer: ‘Have you ever made a purchase through OSM before?’

Interviewee: ‘Yes I did, but I could not do it anymore now. My family has banned me from doing this. I tried it before, I paid the money but I could not get the product’

Interviewer: ‘Have you ever bought something at a higher price (than market price)?’

Interviewee: ‘Yes. Sometimes I need to pay extra, to cover the delivery cost… I did worry about it, therefore I do not do any online purchase anymore (001)

Another male participant had strong interest in playing online games. He mentioned that he had used his brother’s credit card to buy weapons in his game without permission. This has resulted in conflicts between family members when some senior family members sided with him because of his mental condition. These incidents suggested that the unique characteristic of fixated interests among autistic young adults could heighten the risks associated with OSM use. During the interview, he was also reluctant to share any information about the incident and showed irritation when he was asked about further details. This again showed that the traumatic impact of these unpleasant events during OSM use was a critical issue among this group of population.

### Privacy and personal safety related to OSM use

During the interviews, participants repeatedly mentioned common concerns when they used OSM in their daily lives. These included their risk of meeting “bad people” as well as spillage of personal information during OSM use. The worry of meeting “bad people” in OSM was mentioned by five participants. They worried when they were making new friends through online means, that other users may post fake demographic information or photos on their profiles. Some participants also worried that they were unable to differentiate which OSM users were actually scammers who intended to steal their personal information, or seek monetary or sexual gains.

‘I am worried…, I don’t know what kind of person I would meet, sometimes I may meet a bad person, and he may cheat on you, or blackmail you … they may make you take nude photos, or produce other pornographic things… and then they will blackmail you and get your money. They may also threaten you, if you disclose it to police or family, they may put your photos on the Internet’ (010)

Interviewer: ‘When you use OSM, do you have any worry?’

Interviewee: ‘Yes, surely I am worried… I am not sure if others are misrepresenting themselves in their OSM accounts, for example, they may look very attractive in photos, but the real appearance may actually be surprisingly different… it is a kind of cheating …’ (002)

Another concern among our participants was the potential leakage of personal details during their OSM use. Four participants raised their concerns that their own personal ‘details’ could be stolen by other online users. These details were not limited to demographic information such as age, gender, phone number and address, but also included bank account information, personal history, and even their ‘properties’ in online games (such as weapons and coins in games). They worried that their personal information in their user account could be accessed by others without their permission, as well as clicking the links sent by hackers which resulted in them being exploited for information or financial loss.

‘Received some ads about gambling or betting (through OSM platforms)… it required phone number and other personal details, so it was risky if I provided these information’ (003)

Interviewer: I heard that our personal or account details may be leaked, do you worry about the Internet security?

Interviewee: Yes, I did worry that I might have lost my saved data and special collections (in online games)… (004)

When discussing how to protect themselves during OSM use, six participants mentioned that they would avoid talking to strangers during OSM. They expressed that they would not add any strangers as their friends in their OSM accounts, not give any response to the posts written by someone they did not know, and not click on any link which was sent out by a stranger.

‘I won’t add any person whom I don’t know as friend (in OSM account)… and I won’t accept any invitation from strangers as well… this is to ensure my own safety’ (007)

‘I won’t post any comments… only view others’ comments… When I used YouTube, I would not make any friends, and would only read others’ comments, so it should be safe’ (010)

Another three participants said that adhering to Internet security was critically important in protecting their safety during OSM use, which included the use of two-factor authentication and appropriate VPN network safety settings. One participant mentioned the importance of setting a ‘high quality’ password with sufficient length, as well as keeping the password safe.

‘Set passwords… I have three separate accounts, I remember all the passwords which are all very long so that others cannot guess them correctly… besides setting good passwords, we must keep them safely as well’ (003)

## Discussion

The current qualitative study investigated the experiences and risks of OSM use among a group of autistic young adults without intellectual disability in Hong Kong. The results showed while OSM could be a useful platform for young autistic people to connect with friends and broaden their social network, their unique characteristics of autism could make them more vulnerable of unpleasant experiences such as cyberbullying, conflicts with other OSM users and inappropriate online purchasing. Nearly half of our participants reported histories of unpleasant social interactions during their past OSM use. This figure was higher than the prevalence of cyberbullying victimization reported in international findings [31%] (16) as we had employed a broader definition such as witnessing conflicts among other online users. These findings imply that cyberbullying remains a common phenomenon among local autistic young adults which should not be ignored.

The innate difficulties such as poor emotional recognition, a lack of social reciprocity and limited empathetic understanding among autistic people could negatively affect their interactions with friends and create frequent conflicts with others online. For example, their difficulties in understanding *emoji* expressions or hidden meanings of words may create misunderstandings during online communications. Furthermore, they were less able to understand the ‘Internet’ culture and were less aware of the harm caused by their cynical online comments (17) than other young adults. Compared with other young adults, our participants were more likely to take these comments seriously and personally which at times led to their frustration and mood fluctuation. Their difficulties in making friends through online platforms has been similarly reported in another study recruiting adults dating apps users with ASD (6). Their frustration in real-life social situations pushed some autistic participants to actively seek friendships through OSM. Simultaneously, our findings suggested that the high expectation of making new friends could hinder their judgment and put them at risk, such as being cheated on in online social platforms, or developing a high level of trust with online friends in a period of short time due to their strong desire for love and intimacy that may have been missing in their real lives. As reported by one of our participants, one of the worst consequences was the unwanted sexual contact by a friend met through online.

While OSM was meant to facilitate connection with other people, a highly concerning side effect of OSM was the intensified sense of rejection and loneliness. One participant mentioned her frustration when she realized she was excluded from the gatherings of her peer group during OSM use. Previous findings have shown that reduced self-esteem was associated with the sense of being ignored in the OSM activities (16). We, as authors, contend that if their OSM activities provoked feelings of rejection being experienced in real life, these experiences could become re-victimization on line which could compound negative impacts on their self-esteem.

Highly restricted interests which are of abnormal intensity is another core symptom among autistic people. While browsing online discussion forums about their interests offered an opportunity for them to connect with people with similar interests and establish new friendships, conversely their fixated interests and rigidity could create more intense conflicts with other online users. This phenomenon, coupled with poorer mood regulation among autistic people, may result in a lower tolerance to ambiguity or comments expressing opposing viewpoints (e.g., attacking their favorite idols). This in turn could provoke an overreaction to negative posts and reply with aggressive words, which then serve to intensify interpersonal conflicts. On the other hand, due to their inadequate impulse control abilities, their restricted interests also made them less able to resist the temptation of purchasing when preoccupied with specific objects (e.g., weapons in online games). They would try every means to buy what they want from various online forums or social media platforms. This could increase their risk of creating conflicts with family as well as becoming victims of financial scams.

Our findings also suggested that unpleasant events during OSM use could produce exacerbations of traumatic impacts for autistic young adults. Among some of our participants, while these unpleasant events may have occurred many months or years ago, they were still feeling stressed and reluctant to share these events in interviews. During our initial contacts with parents, some shared that OSM was one of the triggers which caused a high level of parenting stress and frequent family conflicts, as well as a deterioration of academic performance and even the need for psychiatric medication to manage mood difficulties among their children. Therefore, effective intervention strategies to promote safe OSM use are urgently needed, and it is crucial for practitioners and researchers to understand the limitations among autistic people, be aware of the potential risks and provide the relevant guidance and support to reduce the harm associated with OSM use.

Despite these risks and downsides, OSM was still viewed by our participants as a helpful tool to connect with other people which allowed “faceless” and non-verbal communication with others. Not only it could help them to maintain connection with their existing friends especially during the COVID-19 pandemic when social gatherings were not recommended, but also offer opportunities for them to meeting new friends with common interests. Furthermore, it provided a more comfortable environment for them to express their inner feelings and thoughts without experiencing the social anxiety which was common among autistic people. These findings supported the ‘additional hypothesis’ (14), meaning that OSM could benefit their interpersonal relationship by supplementing ‘not-so-effective’ real-life communications. These findings also stressed the importance of promoting safe use of OSM and mitigating risks rather than simply adopting a restrictive approach among this group.

Proper privacy and security settings can be effective means to protect the Internet safety among our participants. While some of participants advocated for useful strategies such as firewall settings, and the use of secure passwords as well as OSM account privacy, these were not widely known among all our participants. Therefore, in addition to providing psychoeducation on the topics of making friends and online purchasing, technical knowledge about Internet security could be an important component when designing tailor made intervention to promote safer OSM use among this special group.

### Limitations

Conducting qualitative interviews with autistic people was challenging. A limitation of the current study was the sample size. Recruitment and data collection had been conducted during the peak period of COVID-19 pandemic in Hong Kong and face to face interview was not allowed. Many potential participants refused to join interviews through Zoom as they reported feeling embarrassed and anxious to appear on computer screen. While the reported main themes had been repeatedly identified in our data and saturation was considered to be achieved, the small sample may still limit the richness of our results. As recruiting a random sample was not feasible, the small sample size may also limit how well the results could be generalized to other users in the recruitment sites (DSC and CMS) as well as other local autistic people. In addition, as this research was only conducted in Hong Kong, the current findings may not be able to reflect the situations in Western countries as well. As a result, the findings of current study should be interpreted with the consideration of these limitations.

To maximize participation and create the most comfortable environment for autistic participants to open up and express their thoughts in interviews, their case social workers also carried out the role of interviewers. Potential bias could be possible and should not be ignored, for example, as the participants may feel pressured to join this research due to their pre-existing relationship with case workers, and the role conflicts between case workers and interviewers may affect the flow of interviews. Even with this arrangement, it still took a long warm-up period for our participants to feel relaxed in an ‘interview’ setting. When talking about their previous unpleasant events, they showed much hesitation to share their experiences which could potentially bring traumatic impact. As a result, while the findings provided novel and timely important information regarding their OSM use and experiences, there was a potential underreporting due to their anxiety and stress, and the present study might only reveal a small sub section of the problems encountered by this group.

Despite these limitations, this study was one of the few studies which investigated the OSM use among this unique group of autistic young adults. OSM remains the most universal means to connect with people globally around the world nowadays, however there is still a lack of scientific evidence to show how it can benefit or disadvantage autistic people. Moreover, the current study employed a qualitative study design which enabled an in-depth investigation of the phenomenon of OSM use directly among this vulnerable group. Interview studies with autistic people was in lack in previous research. The uniqueness of this study meant that the qualitative information collected can offer valuable insights into these participants’ habits and needs in order to fill the knowledge gap. This is extremely crucial for designing tailor-made intervention to promote safe OSM use targeting this special group in future. Participatory research can be a future effective approach to engage autistic young adults in the active processes of intervention design and delivery.

## Data availability statement

The raw data supporting the conclusions of this article will be made available by the authors, without undue reservation.

## Ethics statement

The studies involving human participants were reviewed and approved by Institutional Review Board (IRB) of the University of Hong Kong/Hospital Authority Hong Kong West Cluster. The patients/participants provided their written informed consent to participate in this study.

## Author contributions

PL was responsible for the conceptualization, design, data management, and report writing of the research. SL provided expert opinion on the conceptualization and design of the research, as well as critically revised the manuscript. EH provided valuable suggestions on research design, qualitative data collection, and manuscript preparation. CT has contributed to the methodology of research and coordinated different parties to facilitate the data collection process. WW oversaw the whole research, gave advice on the conceptualization and design of the research, data analysis, as well as critically reviewed the manuscript. All authors reviewed and gave final approval to the final manuscript.

## Funding

This study was funded by University Grant Council.

## Conflict of interest

The authors declare that the research was conducted in the absence of any commercial or financial relationships that could be construed as a potential conflict of interest.

## Publisher’s note

All claims expressed in this article are solely those of the authors and do not necessarily represent those of their affiliated organizations, or those of the publisher, the editors and the reviewers. Any product that may be evaluated in this article, or claim that may be made by its manufacturer, is not guaranteed or endorsed by the publisher.

## Figures and Tables

**Table:** 

Experience on the use of online social media (OSM; social platforms, websites or dating apps)
How do you describe your experience of using OSM?
Why do you use these OSM?
How do you think OSM helps you to find your relationship?
Satisfaction of using these OSM and effectiveness of finding friends or potential partners
How do you describe your experience of using OSM to make new friends?
How do you feel when you are using OSM to make friends?
Worries and perception of risks associated with the use of OSM
What worries do you have when you use OSM?
How do you describe your risks associated with using OSM?
Can you describe any unhappy experience when you used OSM?
Measures to protect themselves from possible frauds and spams
What are the possible negative experiences which are associated with the use of OSM?
How do you protect yourself to prevent from the above?
Expectations and how they would benefit from the use of OSM on their social life
How much do you think OSM benefits your social life?
How much do you think OSM help you to start an intimate relationship?
How much do you think OSM benefits your overall quality of life?
In what aspects about OSM which you want to learn more, either to increase its effectiveness or reduce the risks?

## Appendix 1

Questions in the individual interview.
